# Intratumoral Budding in Pretreatment Biopsies, among Tumor Microenvironmental Components, Can Predict Prognosis and Neoadjuvant Therapy Response in Colorectal Adenocarcinoma

**DOI:** 10.3390/medicina58070926

**Published:** 2022-07-12

**Authors:** Kwangil Yim, Won Mo Jang, Uiju Cho, Der Sheng Sun, Yosep Chong, Kyung Jin Seo

**Affiliations:** 1 Department of Hospital Pathology, College of Medicine, The Catholic University of Korea, Seoul 06591, Korea; kangse_manse@catholic.ac.kr (K.Y.); hailtoya@catholic.ac.kr (U.C.); ychong@catholic.ac.kr (Y.C.); 2 Seoul Metropolitan Government—Seoul National University Boramae Medical Center, Department of Public Health and Community Medicine, Seoul 07061, Korea; thomasj@snu.ac.kr; 3 Department of Internal Medicine, College of Medicine, The Catholic University of Korea, Seoul 06591, Korea; medi9652@catholic.ac.kr

**Keywords:** intratumoral budding (ITB), colorectal carcinoma (CRC), tumor microenvironment (TME), desmoplastic reaction (DR), Klintrup–Mäkinen (KM) grade, tumor–stroma ratio (TSR), pretreatment biopsy samples (PBS)

## Abstract

*Background and Objectives:* The prediction of the prognosis and effect of neoadjuvant therapy is vital for patients with advanced or unresectable colorectal carcinoma (CRC). *Materials and Methods*: We investigated several tumor microenvironment factors, such as intratumoral budding (ITB), desmoplastic reaction (DR), and Klintrup–Mäkinen (KM) inflammation grade, and the tumor–stroma ratio (TSR) in pretreatment biopsy samples (PBSs) collected from patients with advanced or unresectable CRC. A total of 85 patients with 74 rectal carcinomas and 11 colon cancers treated at our hospital were enrolled; 66 patients had curative surgery and 19 patients received palliative treatment. *Results:* High-grade ITB was associated with recurrence (*p* = 0.002), death (*p* = 0.034), and cancer-specific death (*p* = 0.034). Immature DR was associated with a higher grade of clinical tumor-node-metastasis stage (cTNM) (*p* = 0.045), cN category (*p* = 0.045), and cM category (*p* = 0.046). The KM grade and TSR were not related to any clinicopathological factors. High-grade ITB had a significant relationship with tumor regression in patients who received curative surgery (*p* = 0.049). *Conclusions:* High-grade ITB in PBSs is a potential unfavorable prognostic factor for patients with advanced CRC. Immature DR, TSR, and KM grade could not predict prognosis or therapy response in PBSs.

## 1. Introduction

Neoadjuvant concurrent chemoradiation therapy (CCRT) has been used as a treatment option for locally advanced rectal carcinoma [1]. Moreover, neoadjuvant chemotherapy has been introduced to manage unresectable colorectal carcinoma (CRC) with oligo-metastasis [2]. This patient group is highly heterogeneous and each patient has a different prognosis [2,3]. To achieve better clinical outcomes, it is necessary to select those who respond well to neoadjuvant treatment. However, considering that radical specimens are deformed because of the neoadjuvant treatment, conventional pathological factors have a limitation for predicting prognosis.

Recently, the tumor microenvironment (TME) has been shown to play a key role in improving treatment or cancer management [4]. Among the TME factors, tumor budding (TB) [5,6,7,8], cancer-associated fibroblasts [9], tumor-infiltrating immune cells [10,11,12], and tumor endothelial cells [4,12] are prognostic markers of various solid cancers. In addition, some factors, such as TB [5,13], peritumoral inflammation [14,15,16,17], and the degree [15,16,18] or features [19,20,21] of fibrosis, are measurable without additional immunohistochemistry, as semi-quantitative or three-tier scoring systems have been developed. In particular, the detection of TB in pretreatment biopsy samples (PBSs), that is, intratumoral budding (ITB) [22,23,24,25,26,27] and CD8-positive T cells [26,27], has been proposed as a predictor of prognosis and/or response to neoadjuvant therapy. However, its prognostic role has not been confirmed and a method of ITB assessment has not been standardized. In addition, other markers for the prediction of prognosis and the response to neoadjuvant therapy are still needed.

In this study, we investigated whether TME factors detected in PBSs using hematoxylin–eosin (HE) staining exclusively can predict prognosis and the neoadjuvant treatment response. For this purpose, we evaluated ITB using various methods that were reported previously and compared their prediction powers regarding survival and therapy response [22,23,24,26,27]. In addition, we classified the type of desmoplastic reaction (DR) as mature, intermediate, or immature [19,20,21]. Finally, we investigated inflammation based on the Klintrup–Mäkinen (KM) grade [14,15,16], and fibrosis was quantified using the tumor–stroma ratio (TSR) [18].

## 2. Materials and Methods

### 2.1. Patient and Clinicopathological Data

We enrolled all patients with CRC who had preoperative treatment (regardless of CCRT or chemotherapy) followed by surgery. A total of 85 patients with 80 rectal carcinomas and 5 colon cancers at Uijeongbu St. Mary’s Hospital between January 2010 and October 2021 were enrolled in this study (Table 1 and Table S1). Among them, 66 patients had curative surgery and 19 patients received palliative treatment (Table S2).

Sixty-three patients (locally advanced rectal cancer (*n* = 58) and rectal cancer with oligometastasis (*n* = 5)) were treated with CCRT. All chemoradiation treatments were CCRT and total 50.4 Gy was divided over 28 days (Table S3). Twenty-two patients with unresectable colon (*n* = 5) and rectal cancer (*n* = 17) had chemotherapy for palliative purpose. Among them, 5 patients had curative surgery with good chemotherapy response, and 17 patients had palliative surgery (Table S4). Details of the CCRT protocol or chemotherapy regimens are summarized in Tables S3 and S4. Clinicopathological parameters, including overall survival (OS), disease-free survival (DFS), cancer-specific survival (CSS), age, sex, tumor location, gross type, size, tumor differentiation, and clinical and pathological tumor-node-metastasis stage (TNM) i.e., cTNM and ypTNM, were retrospectively reviewed from medical records. This study was approved by the Institutional Review Board of the College of Medicine at the Catholic University of Korea (UC21SISI0094).

### 2.2. Histopathological Analyses

**Figure 1:**
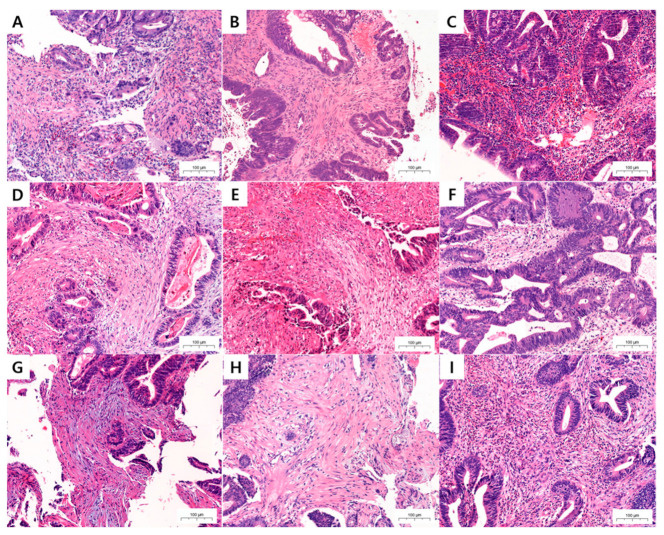
Representative microscopic images of tumor microenvironmental (TME) factors in the pretreatment biopsy samples (PBSs) of colorectal carcinoma (CRC). (**A**) High- and (**B**) low-grade intratumoral budding (ITB). (**C**) Strong and (**D**) weak grade Klintrup–Mäkinen (KM) grade. (**E**) High and (**F**) low ratio of tumor–stromal ratio (TSR). (**G**) Immature, (**H**) intermediate, and (**I**) mature type of desmoplastic reaction (DR). (**A**–**I**, HE staining; 200×).

**Figure 2:**
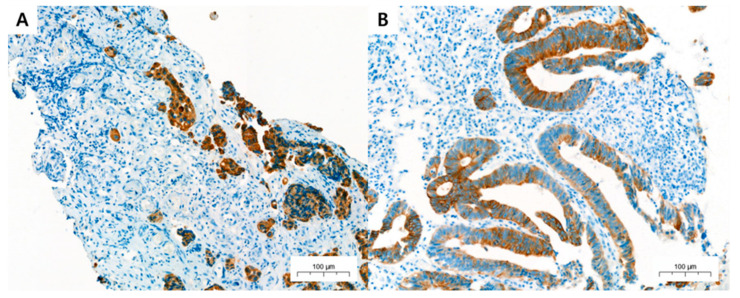
Representative microscopic foci of (**A**) high- and (**B**) low-grade intratumoral budding (ITB) in the pretreatment biopsy samples (PBSs) of colorectal carcinoma (CRC) on pancytokeratin immunohistochemical staining. (**A**,**B**, pancytokeratin AE1/AE3 immunohistochemistry staining; 200×).

HE-stained PBSs were evaluated for neoadjuvant therapy response, ITB, KM grade, TSR, and DR (Figure 1). Neoadjuvant treatment response was evaluated with Dworak classification, and divided into good responder (grades 3 and 4) and poor responder (grades 0, 1, and 2). In addition, ITB was assessed in PBSs using pancytokeratin immunohistochemical staining (IHC) (Figure 2). Two pathologists (K.Y. and K.J.S.) independently assessed all pathological parameters; if any disagreement occurred during this process, it was revolved by consensus, which was reached by meeting with a third pathologist (Y.C.).

### 2.3. Intratumoral Budding

TB has traditionally been defined as isolated single cancer cells or <5 cancer cells in the invasive front [5]. Some authors renamed the traditional TB concept as peritumoral budding and newly defined ITB if TB is detected at the center of the tumor (Figure 1A,B) [5,22].

### 2.4. TB Assessments

First, ITB-total was used to count all TBs in the whole PBS. Second, ITB was classified according to the presence or absence of TB (TB-YN). Third, TB was assessed using the method described by the International Tumor Budding Consensus Conference (ITBCC). After selecting a hotspot by scanning all fields at 100× magnification, we counted the TBs in the selected hotspot within a 200× magnification field. Then, to adjust the TB count to 0.785 mm^2^, the count was converted by applying the normalization factor corresponding to the eyepiece field number [13]. Finally, the ITB×400 method (ITB×400) was used to assess the number of TBs in one hotspot at 400× magnification.

### 2.5. Pancytokeratin Immunohistochemistry

IHC staining was conducted only in 79 patients, because in six cases, the paraffin-embedded blocks were insufficient. Pancytokeratin IHC staining was performed according to the instructions provided in the pathology laboratory manual. Briefly, we obtained unstained slide from paraffin-embedded tissue samples with 3 µm thickness. IHC staining was performed on an automated Ventana Benchmark XT platform (Roche Diagnostics, Basel, Switzerland) using the FDA-approved monoclonal mouse antibody AE1/AE3 clone with additional 4-fold dilution (PA0909, Leica Biosystems, Buffalo Grove, IL, USA) and a Ventana ultraVIEW DAB Detection Kit (Roche Diagnostics, Basel, Switzerland). IHC-stained ITBs (ITB-IHC) were counted using the four methods described above (Figure 2) (ITB-total, ITB-YN, ITB-ITBCC, and ITB×400).

### 2.6. Desmoplastic Reaction Classifications

DRs were classified into immature, intermediate, and mature types [19,20,21]. The immature DR type was selected if myxoid stroma, with a basophilic or amphophilic amorphous mucinous substance, was present. The intermediate DR type was characterized by the absence of a myxoid stroma and the presence of keloid-like collagen, which was observed as a thick bundle of hypocellular collagen with bright eosinophilic hyalinization. Finally, if a myxoid stroma and keloid-like collagen were both absent, the DR was classified into the mature type. The mature DR was composed of multiple fine mature collagen fibers [19,20,21] (Figure 1). We searched all fields with low-power field (LPF), and then selected a hotspot. When myxoid stroma or keloid-like collagen was recognized with certainty, we defined them as being present, regardless of the smallness of their area.

### 2.7. Klintrup–Mäkinen Grade

Originally, the KM grade was designed to evaluate the tumor invasive margin of surgical specimen. However, biopsy samples usually do not include an invasive front; and even if there was one, it would be impossible to detect it in biopsy samples. Therefore, we scanned all fields of the HE slide using LPF, and then selected a hotspot. Finally, we determined the KM grade in a 200× hotspot.

KM grade is scored semi-quantitatively using a four-grade scale. A score of 0 was given when there was no increase in the number of inflammatory cells; a score of 1 denoted a mild and patchy increase in inflammatory cells, but no destruction of invading cancer cell islets by the inflammatory cells; a score of 2 was given when inflammatory cells formed a band-like infiltrate, with some destruction of cancer-cell islets; and a score of 3 denoted a prominent inflammatory reaction, forming a cup-like zone, with frequent destruction of cancer-cell islets [14]. The KM grade is often split into two categories: weak (0 and 1) and strong (2 and 3) [15,16] (Figure 1).

### 2.8. Tumor–Stroma Ratio

The TSR was designed to be scored by assigning a percentage of the proportion of tumor-associated stroma present at the invasive tumor front in a 200× high-power field (HPF) in surgical specimens. We evaluated the TSR in PBSs in the same way as we did the KM grade, which is scanning the all fields then selecting hot spot. The TSR is dichotomized into low (≤50% stroma) and high (>50% stroma) types [16,18] (Figure 1).

### 2.9. Statistical Analyses

The χ^2^ test, Fisher’s exact test, and independent *t*-test were used to compare low- and high-grade ITB, KM grade, TSR, and immature DR. Continuous data were converted to categorical variables using cutoff values, where the sum of sensitivity and specificity was maximized to predict CSS by using a time-dependent receiver operating characteristic (ROC) curve. When performing multivariate Cox regression tests, we used only significantly associated items with prognosis in a Kaplan–Meier curve as a compound factor; then, ITB, KM grade, TSR, and immature DR were added one by one for comparison, to avoid overfitting [28]. When no other factors were significantly associated with prognosis, we used clinical stage as a compound factor. Two-sided *p* values < 0.05 were considered statistically significant. All analyses were performed using the SPSS software (version 20.0; IBM, Armonk, NY, USA) and R statistical programming (version 3.4.1; http//www.r-project.org; accessed on 20 March 2022).

## 3. Results

### 3.1. Clinicopathological Characteristics According to TME Factors in Pretreatment Biopsy Samples

High-grade ITB was associated with recurrence (*p* = 0.002), death (*p* = 0.034), and cancer-specific death (*p* = 0.034). Moreover, immature DR was associated with a higher grade of cN (*p* = 0.045), cM (*p* = 0.046), and cTNM (*p* = 0.045) (Table 1). However, the KM grade and TSR were not correlated with any of the clinicopathological factors (Table S1). Interestingly, high-grade ITB in the whole cohort was not associated with regression (*p* = 0.131); however, high-grade ITB in patients who had received curative surgery exhibited a significant relationship with tumor regression (*p* = 0.049) (Table S2).

### 3.2. Prognostic Value of TME Factors and Multivariate Cox Proportional Hazards Model

**Figure 3:**
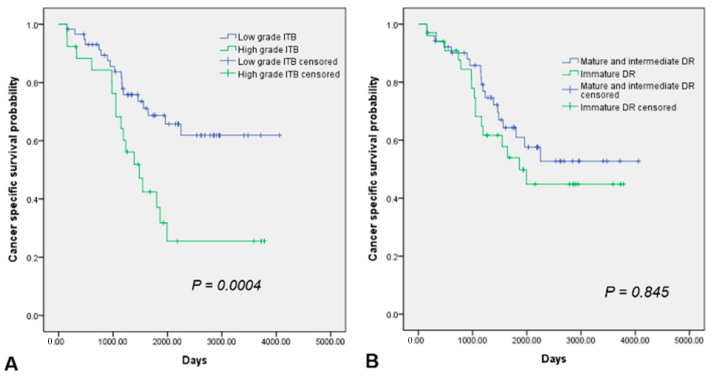
Cumulative cancer-specific survival (CSS) rates in patients with CRC with high and low intratumoral budding (ITB) grades (**A**) and desmoplastic reaction (DR) grades; *p* = 0.0004 for ITB grade (**A**) and *p* = 0.845 for DR grade (**B**).

We found that advanced cT (*p* = 0.013), cM (*p* = 0.002), and high-grade ITB (*p* = 0.005) were poor CSS factors. Moreover, advanced cM (*p* = 0.030) and high-grade ITB (*p* = 0.030) were poor OS factors, and high-grade ITB (*p* < 0.001) was a poor DFS factor exclusively (Table 2). Immature DR, KM grade, and TSR showed no significant association with prognosis (Table 2, Figure 3). In the multivariate analyses, high-grade ITB was an independent poor prognostic factor of CSS (*p* = 0.003) (Table 3, Figure 3).

In subgroup analysis, we also confirmed that ITB was an independent poor prognostic factor (*p* = 0.006) for CSS in the CCRT-treated rectal cancer group, but there was no association with prognosis in the chemotherapy-treated CRC group (*p* = 0.107) (Tables S5 and S6).

### 3.3. Comparison of Predictive Power of ITB Assessment and Staining Methods Using Time-Dependent ROC Curves

The cutoff point of ITB-IHC to maximize the sum of sensitivity and specificity for predicting CSS was >0 for all four assessment methods (ITB-total, ITB-YN, ITB-ITBCC, and ITB×400). The CSS predictive power was the highest in ITB-total among the assessment methods based on HE staining (Table 4). In addition, the predictive power of ITB-IHC was superior to that of ITB-YN, ITB-ITBCC, and ITB×400, but inferior to that of ITB-total (Table 4).

## 4. Discussion

We confirmed that ITB was an independent poor prognostic factor associated with neoadjuvant chemotherapy response in patients with radical surgery. Unfortunately, immature DR, TSR, and KM grade could not predict prognosis or therapy response in PBSs. To the best of our knowledge, this is the first study to investigate whether TME factors without IHC staining can predict prognosis and chemotherapy response in PBSs.

ITB [5,13], immature DR [19,20,21], TSR [18], and KM grade [14,15,16] were originally designed to evaluate the invasive front of radical specimens. However, the biopsy samples usually do not include the invasive margin. This is because the invasive front is located in the deepest point of tumor, whereas the biopsy samples are usually obtained from the superficial part of tumor. The invasive front is the most important area for prognostic determination in various carcinomas [29,30]. In fact, several molecular events related with tumor spread, decreased expression of adhesion molecules, proteolytic enzyme secretion, increased cell proliferation, and initiation of angiogenesis occur at the tumor–host interface, that is, the invasive front [29,30]. In particular, TME factors were originally designed to be measured at the invasive front, because it was assumed that the interaction between tumor and host cells is most active at this location [4,31].

However, some factors showed similar results at the tumor center and invasive front, whereas some factors did not [32,33]. Assessing TB inside the tumor, that is, ITB, yielded results similar to those obtained by evaluating this factor at the invasive front [34]; thus, ITB could be a prognostic biomarker in PBSs [25]. Furthermore, ITB in PBSs can play a role in predicting prognosis and the response to neoadjuvant anticancer therapies [22,23,24,26,27]. Moreover, ITB is correlated with poor clinicopathological factors, such as lymph node metastasis, extranodal tumor deposits, local recurrence, distant metastasis, pT stage, and lymphatic and venous invasion [35,36,37,38,39]. Similarly, we confirmed that high-grade ITB was an independent poor prognostic factor (Table 3) that was significantly associated with ypTNM stage in the whole patient cohort (Table 1).

Unfortunately, immature DR, TSR, and KM grade were not associated with prognosis. This might be because we cannot obtain biopsy samples from the invasive front, where the tumor–host reaction is most active [29,30]. In addition, TSR and KM grade were not associated with any clinicopathological factors, whereas immature DR showed a significant association with a higher grade of cN (*p* = 0.045), cM (*p* = 0.046), and cTNM (*p* = 0.045). Similarly, Shin et al. evaluated the prognostic role and clinicopathological correlation between intratumoral and peritumoral DR and revealed that both intratumoral and peritumoral immature DR were associated with clinicopathological factors [40]. However, intratumoral immature DR could not predict prognosis [40], unlike that observed in other studies that assessed only the peritumoral immature DR [19,21], or evaluated the total tumoral immature DR using a scoring system [20].

We confirmed that ITB was significantly associated with tumor regression in univariate analysis and in the curative surgery group exclusively (*p* = 0.049), but not in multivariate analysis (*p* = 0.056) (Table 1, data not shown). Previously, Rogers et al. classified ITB as being present or absent in 185 patients; however, there was no significant association between ITB and the tumor regression grade [23]. Nevertheless, Chen et al. [26] and Farchoukh et al. [27] counted the number of TBs per 0.785 mm^2^ in 117 patients. They revealed that high-grade ITB (≥2 cells in 0.785 mm^2^) was significantly associated with tumor regression in univariate [26,27] and multivariate [27] analyses. Given the small number of studies and their relatively small number of included patients, further studies are still warranted to confirm the predictive power of ITB for neoadjuvant therapy.

In addition, we revealed that the predictive value of ITB was only effective in the curative surgery group (*p* = 0.049), because when the palliative surgery group was included in the analysis, there was no association between ITB and tumor regression (*p* = 0.131) (Table 1 and Table S2). TB is associated with tumor-cell dissociation, invasion, and migration [5]. Therefore, in the setting of advanced metastatic stage, TB could be less predictive regarding prognosis and/or the chemotherapy response.

We revealed that the predictive power of TB-IHC was similar or inferior to that of the assessment using HE staining (Table 4). The advantage of IHC was that it showed TB more clearly and reduced subjectivity [13,41]. However, it also stained apoptotic tumor cells; therefore, recent studies have shown no significant difference between HE and IHC [13,42]. Therefore, the ITBCC recommended that HE staining be used as a routine method, and that IHC should be used when the assessment was difficult, such as in the presence of severe background inflammation [13]. However, there is no guideline for PBSs. Most studies used HE staining [22,23,24,25,26,27,35,36,37,38] and only one study used pancytokeratin IHC [39]; moreover, there are no studies comparing the two staining methods. We also suggest the use of HE staining as a routine method, because of its lower cost and similarity to ITBCC recommendations in radical specimens [13].

Moreover, we directly compared several ITB assessment methods; subsequently, we revealed that ITB-total had a superior prognosis predictive power compared with TB-ITBCC, TB-YN, and ITB×400. TB-YN is simple and highly reproducible [8]; however, TB mimickers, such as macrophages, tangentially sectioned tumor glands, and apoptotic tumor cells, can be misinterpreted as TB cells. Using TB-ITBCC and TB×400, the number of TBs was counted in only one hotspot. However, in biopsy samples, the size of the lesion is usually insufficient for representation of the whole tumor. Therefore, we need to collect all information contained in biopsy samples. In addition, counting the overall number of TBs is less laborious when the cutoff value (in this study, ≥5 tumor buds) is low.

Previously, TB-YN [23,38], a hotspot with 200× magnification (TB×200) [36,37], TB×400 [35], or TB-ITBCC [26,27] was used for TB assessment with, usually, a cutoff value < 5 cells. In these previous studies, researchers scanned the whole specimen with a low-power field, then selected one hotspot with 200× (TB×200) or 400× (TB×400) magnification. Then, they counted the number of TBs in the hotspot. In the TB-ITBCC method, the number of TBs was converted to a count in 0.785 mm^2^. Although most studies showed similar poor prognostic results, in real practice, we still need a well-organized scoring system affiliated with the reputed committee for standardization. Here, we directly compared several methods, then recommended TB-total as a standard; however, further studies using a larger population with a reputed committee are still needed.

Our study had several limitations, especially the relatively low sample number (statistical power = 0.800); therefore, further studies on a larger scale, especially in patients with neoadjuvant-chemotherapy-treated CRC, are required. However, as we enrolled patients with CRC who were treated with the same regimen (neoadjuvant chemotherapy followed by surgery) at one institution over 10 years, our study cohort may reflect the results of real-world practice.

In addition, the CRC cohort in this study was heterogeneous in terms of treatment options (CCRT or chemotherapy). We wanted to find out the predictive role of TME factors in PBSs for prognosis and treatment response not only in patients with locally advanced rectal cancers, but also in patients with unresectable colon cancers treated with chemotherapy followed by surgery. The number of patients with preoperative treatment enrolled was relatively small (especially in chemotherapy group), thus we tried to keep a balance that preserved group homogeneity while selecting as many patients as possible. Considering that previous studies also reported that ITB could predict prognosis in CRC and that most CRC share the same pathogenesis, this inclusion criterion could be clinically relevant. Furthermore, we conducted multivariate Cox proportional hazards analysis for predicting CSS, with adding treatment option as a compounding factor, to adjust treatment option heterogeneity. Therefore, we confirmed that ITB was an independent prognostic factor (*p* = 0.003) (Table 3).

Finally, interobserver variation may exist in the interpretation of ITB, TSR, KM grade, and DR. To overcome this limitation, a high grade of each of the factors was only recognized if the morphology was determined based on the agreement of three pathologists (K.Y., Y.C. and K.J.S.). When there was a disagreement between the pathologists, it was resolved by the consensus of the three pathologists.

## 5. Conclusions

In conclusion, we confirmed that high-grade ITB in PBSs is a potential unfavorable prognostic factor for patients with advanced CRC. Unfortunately, other TME factors, that is, KM grade, TSR, and immature DR, were not associated with prognosis or treatment response in biopsy samples. In addition, we revealed that the predictive prognostic power of ITB-IHC was similar or inferior to that of the assessment methods based on HE staining. We also compared several ITB assessment methods and revealed that ITB-total was superior to TB-ITBCC, TB-YN, and TB×400 for the prediction of prognosis. These findings may contribute to treatment options for CRC patients who need neoadjuvant chemotherapy.

## Figures and Tables

**Table 1:** Comparison of clinicopathological and histological parameters, intratumoral budding (ITB), and desmoplastic reaction (DR) between low- and high-grade ITB and mature, intermediate and immature DR in pretreatment biopsy samples (PBSs).

		ITB			DR		
		Low-Grade (*n* = 59)	High-Grade (*n* = 26)	*p* Value	Mature + Intermediate (*n* = 51)	Immature (*n* = 34)	*p* Value
Age	Years (mean ± SD)	64.05 ± 10.45	62.46 ± 8.82	0.501	63.14 ± 10.11	64.21 ± 9.83	0.631
Sex	Male	39 (70.9%)	16 (29.1%)	0.685	35 (63.6%)	20 (36.4%)	0.354
	Female	20 (66.7%)	10 (33.3%)		16 (53.3%)	14 (46.7%)	
Site	Right-sided	3 (60.0%)	2 (40.0%)	0.722	3 (60.0%)	2 (40.0%)	0.714
	Left-sided	1 (100.0%)	0 (0.0%)		1 (100.0%)	0 (0.0%)	
	Rectal	55 (69.6%)	24 (30.4%)		47 (59.5%)	32 (40.5%)	
Surgery	Curative	47 (71.2%)	19 (28.8%)	0.575	43 (65.2%)	23 (34.8%)	0.110
	Palliative	12 (63.2%)	7 (36.8%)		8 (42.1%)	11 (57.9%)	
cT	T2 + T3	35 (76.1%)	11 (23.9%)	0.147	29 (63.0%)	17 (37.0%)	0.534
	T4	24 (61.5%)	15 (38.5%)		22 (56.4%)	17 (43.6%)	
cN	N0	8 (80.0%)	2 (20.0%)	0.717	9 (90.0%)	1 (10.0%)	0.045 *
	N1 + N2	51 (68.0%)	24 (32.0%)		42 (56.0%)	33 (44.0%)	
cM	M0	42 (72.4%)	16 (27.6%)	0.379	39 (67.2%)	19 (32.8%)	0.046 *
	M1	17 (63.0%)	10 (37.0%)		12 (44.4%)	15 (55.6%)	
cTNM	I + II	8 (80.0%)	2 (20.0%)	0.717	9 (90.0%)	1 (10.0%)	0.045 *
	III + IV	51 (68.0%)	24 (32.0%)		42 (56.0%)	33 (44.0%)	
ypT	Tis + T1 + T2	19 (86.4%)	3 (13.6%)	0.060	16 (72.7%)	6 (27.3%)	0.209
	T3 + T4	40 (63.5%)	23 (36.5%)		35 (55.6%)	28 (44.4%)	
ypN	N0	42 (76.4%)	13 (23.6%)	0.060	36 (65.5%)	19 (34.5%)	0.165
	N1 + N2	17 (56.7%)	13 (43.3%)		15 (50.0%)	15 (50.0%)	
ypM	M0	43 (72.9%)	16 (27.1%)	0.296	40 (67.8%)	19 (32.2%)	0.033 *
	M1	16 (61.5%)	10 (38.5%)		11 (42.3%)	15 (57.7%)	
ypTNM	I + II	38 (80.9%)	9 (19.1%)	0.017 *	33 (70.2%)	14 (29.8%)	0.033 *
	III + IV	21 (55.3%)	17 (44.7%)		18 (47.4%)	20 (52.6%)	
Recurrence	No	47 (79.7%)	12 (20.3%)	0.002 *	36 (61.0%)	23 (39.0%)	0.773
	Yes	12 (46.2%)	14 (53.8%)		15 (57.7%)	11 (42.3%)	
Death	No	34 (81.0%)	8 (18.0%)	0.034 *	27 (64.3%)	15 (35.7%)	0.425
	Yes	25 (58.1%)	18 (41.9%)		24 (55.8%)	19 (44.2%)	
CSD	No	34 (81.0%)	8 (19.0%)	0.034 *	27 (64.3%)	15 (35.7%)	0.425
	Yes	25 (58.1%)	18 (41.9%)		24 (55.8%)	19 (44.2%)	
Regression	Good	14 (87.5%)	2 (12.5%)	0.131	10 (62.5%)	6 (37.5%)	1.000
	Poor	45 (65.2%)	24 (34.8%)		41 (59.4%)	28 (40.6%)	
Treatment	CCRT	44(69.8%)	19 (30.2%)	1.000	42 (66.7%)	21 (33.3%)	0.044 *
	CTx	15 (68.2%)	7 (31.8%)		9 (40.9%)	13 (59.1%)	

Data are presented as *n* (%), mean ± SD, and median (range). The *p* value of significant differences between present/absent LMN was obtained by χ^2^, Fisher’s exact, and independent *t*-tests. c, clinical; T, T category; N, N category; M, M category; TNM, tumor-node-metastasis stage; CSD, cancer-specific death; ITB, intratumoral budding; DR, desmoplastic reaction; yp, post-treatment pathologic; CCRT, concurrent chemoradiation therapy; CTx, chemotherapy; * statistically significant.

**Table 2:** The prognostic significances of the TME factors, such as ITB, DR, TSR, and KM grade in PBSs using the Kaplan–Meier curve analysis.

	DFS (*p* Value)	OS (*p* Value)	CSS (*p* Value)
Age	0.665	0.675	0.601
Sex	0.263	0.163	0.090
cT	0.106	0.055	0.013 *
cN	0.896	0.430	0.726
cM	0.973	0.030 *	0.002 *
cTNM	0.896	0.430	0.726
Treatment	0.922	0.016 *	0.001 *
TME factors			
ITB	<0.001 *	0.030 *	0.005 *
DR	0.264	0.422	0.359
TSR	0.127	0.385	0.165
KM	0.888	0.755	0.930

TME, tumor microenvironment; ITB, intratumoral budding; DR, desmoplastic reaction; TSR, tumor–stroma ratio; KM, Klintrup–Mäkinen grade; c, clinical; T, T category; N, N category; M, M category; TNM, tumor-node-metastasis stage; DFS, disease-free survival; OS, overall survival; CSS, cancer-specific survival; * statistically significant.

**Table 3:** Multivariate Cox proportional hazards analysis of predictors for CSS.

	HR (95% CI)	*p* Value
cT	1.263 (0.673–2.373)	0.467
cM	1.533 (0.355–6.622)	0.567
Treatment	1.972 (0.450–8.645)	0.368
TME factors		
ITB *	2.829 (1.437–5.571)	0.003 ^†^
DR *	1.044 (0.524–2.081)	0.902
KM *	0.715 (0.555–2.360)	0.715
TSR *	0.912 (0.437–2.571)	0.853

CSS, cancer-specific survival; HR, hazard ratio; CI, confidence interval; c, clinical; T, T category; N, N category; M, M category; TNM, tumor-node-metastasis stage; TME, tumor microenvironment; ITB, intratumoral budding; DR, desmoplastic reaction; KM, Klintrup–Mäkinen grade; TSR, tumor–stroma ratio; * adjusted for cT, cM, and treatment; ^†^ statistically significant.

**Table 4:** Performance of two methods of assessing TB based on HE and pancytokeratin IHC.

	HE (*n* = 85)		HE and IHC-PK (*n* = 79)	
	AUC	Difference AUC	AUC	Difference AUC
Crude	0.648		0.618	
TB-total	0.711	0.063	0.691	0.073
TB-ITBCC	0.679	0.031	0.649	0.031
TB×400	0.677	0.029	0.651	0.033
TB-YN	0.679	0.031	0.649	0.031
TB-IHC			0.679	0.061

TB, Tumor budding; HE, hematoxylin–eosin stain; IHC-PK, immunohistochemical staining of pancytokeratin; AUC, area under the curve; ITBCC, International Tumor Budding Consensus Conference; TB-YN, presence or absence of TB.

## Data Availability

The datasets used and analyzed during the current study are available from the corresponding author on reasonable request.

## Supplementary Materials

The following supporting information can be downloaded at: https://www.mdpi.com/article/10.3390/medicina58070926/s1, Table S1: Comparison of clinicopathological factors and KM grade or TSR; Table S2: Correlation of high-grade ITB and good tumor regression in the cohort of CRC patients who had received curative surgery; Table S3: Associations between factors of the TME, such as ITB, DR, KM, and TSR in PBSs and various clinicopathological features in the cohort of colorectal cancer patients who received neoadjuvant chemoradiotherapy; Table S4: Associations between ITB, DR, and KM in PBSs and various clinicopathological features in the subgroup of colorectal cancer patients who received chemotherapy; Table S5: The prognostic significances of the TME factors, such as ITB, DR, TSR, and KM grade in PBSs using the Kaplan–Meier curve analysis in two subgroups (CCRT vs. CTx); Table S6: Multivariate Cox proportional hazards analysis of predictors for CSS in the CCRT subgroup.
