# Supplementary material for: Intratumoral Budding in Pretreatment Biopsies, among Tumor Microenvironmental Components, Can Predict Prognosis and Neoadjuvant Therapy Response in Colorectal Adenocarcinoma
Source: Medicina (Kaunas). 2022 Jul 12;58(7):926. doi: 10.3390/medicina58070926 (PMC9324564; doi:10.3390/medicina58070926)
Supplement: Supplementary file 1 [file medicina-58-00926-s001.zip › medicina-1793260-supplementary.pdf]

Table S1. Comparison of clinicopathological factors and KM or TSR in the colorectal cancer cohort.

factors		KM			TSR		
		Low-grade n = 58	High-grade n = 27	*P* value	≤50% n = 6	>50% n = 79	*P* value
Age	Years old (means ± SD)	64.50 ± 10.44	61.56 ± 8.67	0.206	70.00 ± 10.86	63.08 ± 9.78	0.101
Sex	Male	37 (67.3%)	18 (32.7%)	1.000	3 (5.5%)	52 (94.5%)	0.661
	Female	21 (70.0%)	9 (30.0%)		3 (10.0%)	27 (90.0%)	
Site	Colon	5 (100.0%)	0 (0.0%)	0.173	0 (0.0%)	5 (100.0%)	1.000
	Rectum	53 (66.2%)	27 (33.8%)		6 (7.5%)	74 (92.5%)	
Surgery	Curative	46 (69.7%)	20 (30.3%)	0.588	6 (9.1%)	60 (90.9%)	0.330
	Palliative	12 (63.2%)	7 (36.8%)		0 (0.0%)	19 (100.0%)	
cT	T2 + T3	31 (67.4%)	15 (32.6%)	0.856	5 (10.9%)	41 (89.1%)	0.212
	T4	27 (69.2%)	12 (30.8%)		1 (2.6%)	38 (97.4%)	
cN	N0	7 (70.0%)	3 (30.0%)	1.000	1 (10.0%)	9 (90.0%)	0.540
	N1 + N2	51 (68.0%)	24 (32.0%)		5 (6.7%)	70 (93.3%)	
cM	M0	39 (67.2%)	19 (32.8%)	0.808	6 (10.3%)	52 (89.7%)	0.170
	M1	19 (70.4%)	8 (29.6%)		0 (0.0%)	27 (100.0%)	
cTNM	I+II	7 (70.0%)	3 (30.0%)	1.000	1 (10.0%)	9 (90.0%)	0.540
	III+IV	51 (68.0%)	24 (32.0%)		5 (6.7%)	70 (93.3%)	
ypT	Tis+T1+T2	16 (72.7%)	6 (27.3%)	0.791	3 (13.6%)	19 (86.4%)	0.176
	T3+T4	42 (66.7%)	21 (33.3%)		3 (4.8%)	60 (95.2%)	
ypN	N0	36 (65.5%)	19 (23.6%)	0.456	4 (7.3%)	51 (92.7%)	1.000
	N1+N2	22 (73.3%)	8 (26.7%)		2 (6.7%)	28 (93.3%)	
ypM	M0	40 (67.8%)	19 (32.2%)	1.000	5 (8.5%)	54 (91.5%)	0.662
	M1	18 (69.2%)	8 (30.8%)		1 (3.8%)	25 (96.2%)	
ypTNM	I+II	32 (68.1%)	15 (31.9%)	0.974	4 (8.5%)	43 (91.5%)	0.687
	III+IV	26 (68.4%)	12 (31.6%)		2 (5.3%)	36 (94.7%)	
CSD	No	34 (68.0%)	16 (32.0%)	0.956	2 (4.0%)	48 (96.0%)	0.224
	Yes	24 (68.6%)	11 (31.4%)		4 (11.4%)	31 (88.6%)	
Death	No	27 (64.3%)	15 (35.7%)	0.440	2 (4.8%)	40 (95.2%)	0.676
	Yes	31 (72.1%)	12 (27.9%)		4 (9.3%)	39 (90.7%)	
Recurrence	No	39 (66.1%)	20 (33.9%)	0.618	2 (3.4%)	57 (96.6%)	0.068
	Yes	19 (73.1%)	7 (53.8%)		4 (15.4%)	22 (84.6%)	
Regression	Good	47 (68.1%)	22 (31.9%)	1.000	5 (7.2%)	64 (92.8%)	1.000
	Poor	11 (68.8%)	5 (31.2%)		1 (6.2%)	15 (93.8%)	
Treatment	CCRT	42 (66.7%)	21 (33.3%)	0.791	6 (9.5%)	57 (90.5%)	0.332
	CTx	16 (72.7%)	6 (27.3%)		0 (0.0%)	22 (100.0%)	

Data are presented as n (%) and as the mean ± SD, median (range). The *P* value of significant difference s between present/absent LMN was obtained by χ^2, Fisher's exact, and independent *t*-tests. KM, Klintrup-Mäkinen grade; TSR, Tumor stroma ratio; c-, clinical; T, T category; N, N category; M, M category; TNM, tu mor-node-metastasis stage; yp-; post-treatment pathologic ; CSD, cancer-specific death; ITB, intratumoral bud ding; DR, desmoplastic reaction; CCRT, concurrent chemoradiation therapy; CTx, chemotherapy.

Table S2. Association between TME factors such as ITB, DR, KM, TSR and clinicopathological factors in the cohort of rectal cancer patients received curative surgery after neoadjuvant chemoradiotherapy.

		ITB			DR			KM grade			TSR		
		Low-grade n = 47	High-grade n = 19	*P* value	Mature + intermediate n = 43	Immature n = 23	*P* value	Low-grade n = 46	High-grade n = 20	*P* value	≤50% n = 6	>50% n = 60	*P* value
Age	years old (mean ± SD)	65.45 ± 9.97	61.89 ± 9.90	0.448	63.56 ± 9.83	64.04 ± 10.34	0.340	65.33 ± 10.26	62.35 ± 9.31	0.270	70.00 ± 10.86	63.87 ± 9.84	0.154
Sex	Male	31 (70.5%)	13 (29.5%)	1.000	30 (68.2%)	14 (31.8%)	0.585	31 (70.5%)	13 (29.5%)	1.000	3 (6.8%)	41 (93.2%)	0.392
	Female	16 (72.7%)	6 (27.3%)		13 (59.1%)	9 (40.9%)		15 (68.2%)	7 (31.8%)		3 (13.6%)	19 (86.4%)	
cT	T2 + T3	32 (76.2%)	10 (23.8%)	0.268	28 (66.7%)	14 (33.3%)	0.792	29 (69.0%)	13 (31.0%)	1.000	5 (11.9%)	37 (88.1%)	0.404
	T4	15 (62.5%)	9 (37.5%)		15 (62.5%)	9 (37.5%)		17 (70.8%)	7 (29.2%)		1 (4.2%)	23 (95.8%)	
cN	N0	8 (80.0%)	2 (20.0%)	0.711	9 (90.0%)	1 (10.0%)	0.146	7 (70.0%)	3 (30.0%)	0.982	1 (10.0%)	9 (90.0%)	1.000
	N1 + N2	39 (69.6%)	17 (30.4%)		34 (60.7%)	22 (39.3%)		39 (69.6%)	17 (30.4%)		5 (8.9%)	51 (91.1%)	
cM	M0	42 (72.4%)	16 (27.6%)	0.680	39 (67.2%)	19 (32.8%)	0.435	39 (67.2%)	19 (32.8%)	0.418	6 (10.3%)	52 (89.7%)	1.000
	M1	5 (62.5%)	3 (37.5%)		4 (50.0%)	4 (50.0%)		7 (87.5%)	1 (12.5%)		0 (0%)	8 (100.0%)	
cTNM	I + II	8 (80.0%)	2 (20.0%)	0.711	9 (90.0%)	1 (10.0%)	0.146	7 (70.0%)	3 (30.0%)	0.982	1 (10.0%)	9 (90.0%)	1.000
	III + IV	39 (69.6%)	17 (30.4%)		34 (60.7	22 (39.3%)		39 (69.6%)	17 (30.4%)		5 (8.9%)	51 (91.1%)	
ypT	Tis + T1 + T2	19 (90.5%)	2 (9.5%)	0.021	16 (76.2%)	5 (23.8%)	0.270	15 (71.4%)	6 (28.6%)	1.000	3 (14.3%)	18 (85.7%)	0.373
	T3 + T4	28 (62.2%)	17 (37.8%)		27 (60.0%)	18 (40.0%)		31 (68.9%)	14 (31.1%)		3 (6.7%)	42 (93.3%)	
ypN	N0	39 (78.0%)	11 (22.0%)	0.054	34 (68.0%)	16 (32.0%)	0.547	34 (68.0%)	16 (32.0%)	0.758	4 (8.0%)	46 (92.0%)	0.627
	N1 + N2	8 (50.0%)	8 (50.0%)		9 (56.2%)	7 (43.8%)		12 (75.0%)	4 (25.0%)		2 (12.5%)	14 (87.5%)	
ypM	M0	43 (74.1%)	15 (25.9%)	0.213	40 (69.0%)	18 (31.0%)	0.115	39 (67.2%)	19 (32.8%)	0.418	5 (8.6%)	53 (91.4%)	0.555
	M1	4 (50.0%)	4 (50.0%)		3 (37.5%)	5 (62.5%)		7 (87.5%)	1 (12.5%)		1 (12.5%)	7 (87.5%)	
ypTNM	I + II	38 (80.9%)	9 (19.1%)	0.014	33 (70.2%)	14 (29.8%)	0.254	32 (68.1%)	15 (31.9%)	0.772	4 (8.5%)	43 (91.5%)	1.000
	III + IV	9 (47.4%)	10 (52.6%)		10 (52.6%)	9 (47.4%)		14 (73.7%)	5 (26.3%)		2 (10.5%)	17 (89.5%)	
CSD	No	37 (82.2%)	8 (17.8%)	0.007	29 (64.4%)	16 (35.6%)	1.000	31 (68.9%)	14 (31.1%)	1.000	2 (4.4%)	43 (95.6%)	0.076
	Yes	10 (47.6%)	11 (52.4%)		14 (66.7%)	7 (33.3%)		15 (71.4%)	6 (28.6%)		4 (19.0%)	17 (81.0%)	
Death	No	30 (81.1%)	7 (18.9%)	0.058	24 (64.9%)	13 (35.1%)	0.956	24 (64.9%)	13 (35.1%)	0.422	2 (5.4%)	35 (94.6%)	0.392
	Yes	17 (58.6%)	12 (41.4%)		19 (65.5%)	10 (34.5%)		22 (75.9%)	7 (24.1%)		4 (13.8%)	25 (86.2%)	
Recurrence	No	35 (87.5%)	5 (12.5%)	0.001	28 (70.0%)	12 (30.0%)	0.305	27 (67.5%)	13 (32.5%)	0.785	2 (5.0%)	38 (95.0%)	0.202
	Yes	12 (46.2%)	14 (53.8%)		15 (57.7%)	11 (42.3%)		19 (73.1%)	7 (26.9%)		4 (15.4%)	22 (84.6%)	
Regression	Good	14 (93.3%)	1 (6.7%)	0.049	10 (66.7%)	5 (33.3%)	1.000	10 (66.7%)	5 (33.3%)	0.759	1 (6.7%)	14 (93.3%)	1.000
	Poor	33 (64.7%)	18 (35.3%)		33 (64.7%)	18 (35.3%)		36 (70.6%)	15 (29.4%)		5 (9.8%)	46 (90.2%)	

ITB, intratumoral budding; DR, desmoplastic reaction; KM, Klintrup–Mäkinen grade; TSR, tumor–stroma ratio; c-, clinical; T, T category; N, N category; M, M category; TNM, tumor-node-metastasis stage; yp-; post-treatment pathologic; CSD, cancer-specific death.

Table S3. Associations between factors of the TME, such as ITB, DR, KM and TSR in PBSs and various clinicopathological features in the cohort of colorectal cancer patients received neoadjuvant chemoradiotherapy.

		ITB			DR			KM			TSR		
		Low-grade (number, %)	High-grade (number, %)	*P* value	Mature intermediate (number, %)	Immature (number, %)	*P* value	Low-grade (number, %)	High-grade (number, %)	*P* value	≤50%	>50%	*P* value
Chemotherapy regimen	LF	27 (73.0%)	10 (27.0%)		22 (59.5%)	15 (40.5%)		23 (62.2%)	14 (37.8%)		2 (5.4%)	35 (94.6%)	
	capecitabine	14 (63.6%)	8 (36.4%)	0.732	17 (77.3%_	5 (22.7%)	0.349	16 (72.7%)	6 (27.3%)	0.662	4 (18.2%)	18 (81.8%)	0.216
	FOLFOX	3 (75.0%)	1 (25.0%)		3 (75.0%)	1 (25.0%)		3 (75.0%)	1 (25.0%)		0 (0.0%)	4 (100.0%)	
Age	years old (means ± SD)	65.70 ± 9.71	62.58 ± 9.60	0.244	63.81 ± 9.42	66.67 ± 10.22	0.274	66.00 ± 9.88	62.29 ± 9.08	0.154	70.00 ± 10.86	64.21 ± 9.52	0.167
Sex	Male	30 (71.4%)	12 (28.6%)	0.774	30 (71.4%)	12 (28.6%)	1.000	28 (66.7%)	14 (33.3%)	1.000	3 (7.1%)	39 (92.9%)	0.391
	Female	14 (66.7%)	7 (33.3%)		12 (57.1%)	9 (42.9%)		14 (66.7%)	7 (33.3%)		3 (14.3%)	18 (85.7%)	
cT	T2+T3	30 (78.9%)	8 (21.1%)	0.091	27 (71.1%)	11 (28.9%)	0.363	25 (65.8%)	13 (34.2%)	1.000	5 (13.2%)	33 (86.8%)	0.389
	T4	14 (56.0%)	11 (44.0%)		15 (60.0%)	10 (40.0%)		17 (68.0%)	8 (32.0%)		1 (4.0%)	24 (96.0%)	
cN	N0	8 (80.0%)	2 (20.0%)	0.711	9 (90.0%)	1 (10.0%)	0.144	7 (70.0%)	3 (30.0%)	1.000	1 (10.0%)	9 (90.0%)	1.000
	N1+N2	36 (67.9%)	17 (32.1%)		33 (62.3%)	22 (37.7%)		35 (66.0%)	18 (34.0%)		5 (9.4%)	48 (90.6%)	
cM	M0	42 (72.4%)	16 (27.6%)	0.156	39 (67.2%)	19 (32.8%)	1.000	39 (67.2%)	19 (32.8%)	1.000	6 (10.3%)	52 (89.7%)	1.000
	M1	2 (40.0%)	3 (60.0%)		3 (60.0%)	2 (40.0%)		3 (60.0%)	2 (40.0%)		0 (0%)	5 (100.0%)	
cTNM	I+II	8 (80.0%)	2 (20.0%)	0.709	9 (90.0%)	1 (10.0%)	0.144	7 (70.0%)	3 (30.0%)	1.000	1 (10.0%)	9 (90.0%)	1.000
	III+IV	36 (67.9%)	17 (32.1%)		33 (62.3%)	20 (37.7%)		35 (66.0%)	18 (34.0%)		5 (9.4%)	48 (90.6%)	
ypT	Tis+T1+T2	19 (86.4%)	3 (13.6%)	0.046	16 (72.7%)	6 (27.3%)	0.578	16 (72.7%)	6 (27.3%)	0.578	3 (13.6%)	19 (86.4%)	0.413
	T3+T4	25 (61.0%)	16 (39.0%)		26 (63.4%)	15 (36.6%)		26 (63.4%)	15 (26.6%)		3 (7.3%)	38 (92.7%)	
ypN	N0	37 (77.1%)	11 (22.9%)	0.050	33 (68.8%)	15 (31.2%)	0.545	32 (66.7%)	16 (33.3%)	1.000	4 (8.3%)	44 (91.7%)	0.622
	N1+N2	7 (46.7%)	8 (53.3%)		9 (60.0%)	6 (40.0%)		10 (66.7%)	5 (33.3%)		2 (13.3%)	13 (86.7%)	
ypM	M0	42 (73.7%)	15 (26.3%)	0.062	39 (68.4%)	18 (31.6%)	0.391	38 (66.7%)	19 (33.3%)	0.418	5 (8.8%)	52 (91.2%)	0.466
	M1	2 (33.3%)	4 (66.7%)		3 (50.0%)	3 (50.0%)		4 (66.7%)	2 (33.3%)		1 (16.7%)	5 (83.3%)	
ypTNM	I+II	37 (80.4%)	9 (19.6%)	0.005	32 (69.6%)	14 (30.4%)	0.549	31 (67.4%)	15 (32.6%)	1.000	4 (8.7%)	42 (91.3%)	0.657
	III+IV	7 (41.2%)	10 (58.8%)		10 (58.8%)	7 (41.2%)		11 (64.7%)	6 (35.3%)		2 (11.8%)	15 (88.2%)	
CSD	No	35 (81.4%)	8 (18.6%)	0.007	28 (65.1%)	15 (34.9%)	0.780	28 (65.1%)	15 (34.9%)	0.780	2 (4.7%)	41 (95.3%)	0.075
	Yes	9 (45.0%)	11 (55.0%)		14 (70.0%)	6 (30.0%)		14 (70.0%)	6 (30.0%)		4 (20.0%)	16 (80.0%)	
Death	No	28 (80.0%)	7 (20.0%)	0.059	23 (65.7%)	12 (34.3%)	1.000	21 (60.0%)	14 (40.0%)	0.284	2 (5.7%)	33 (94.3%)	0.393

	Yes	16 (57.1%)	12 (42.9%)		19 (67.9%)	9 (32.1%)		21 (75.0%)	7 (25.0%)		4 (14.3%)	24 (85.7%)	
Recurrence	No	33 (84.6%)	6 (15.4%)	0.002	27 (69.2%)	12 (30.8%)	0.595	25 (64.1%)	14 (35.9%)	0.784	2 (5.1%)	37 (94.9%)	0.190
	Yes	11 (45.8%)	13 (54.2%)		15 (62.5%)	9 (37.5%)		17 (70.8%)	7 (29.2%)		4 (16.7%)	20 (83.3%)	
Regression	Good	14 (87.5%)	2 (12.5%)	0.115	10 (62.5%)	6 (37.5%)	0.762	11 (68.8%)	5 (31.2%)	1.000	1 (6.2%)	15 (93.8%)	1.000
	Poor	30 (63.8%)	17 (36.2%)		32 (68.1%)	15 (31.9%)		36 (66.0%)	16 (34.0%)		5 (10.6%)	42 (89.4%)	

TME, tumor microenvironment; ITB, intratumoral budding; DR, desmoplastic reaction; KM, Klintrup–Mäkinen grade; TSR, tumor–stroma ratio; LF, leucovorin and 5-fluorouracil combination; FOLFOX, leucovorin, 5-fluorouracil and oxaliplatin combination; c-, clinical; T, T category; N, N category; M, M category; TNM, tumor-node-metastasis stage; yp-; post-treatment pathologic ; CSD, cancer-specific death

Table S4. Associations between ITB, DR, and KM in PBSs and various clinicopathological features in the subgroup of colorectal cancer patients received chemotherapy.

		ITB			DR			KM		
		Low-grade n = 15	High-grade n = 7	*P* value	Mature + intermediate n = 9	Immature n = 13	*P* value	Low-grade n = 6	High-grade n = 6	*P* value
Chemotherapy regimen	FOLFIRI + bevacizumab	4 (50.0%)	4 (50.0%)	0.434	2 (25.0%)	6 (75.0%)	0.259	6 (75.0%)	2 (25.0%)	0.993
	FOLFIRI + cetuximab	5 (71.4%)	2 (28.6%)		5 (71.4%)	2 (28.6%)		5 (71.4%)	2 (28.6%)	
	FOLFOX	3 (100.0%)	0 (0.0%)		1 (33.3%)	2 (66.7%)		2 (66.7%)	1 (33.3%)	
	FOLFIRI	3 (75.0%)	1 (25.0%)		1 (25.0%)	3 (75.0%)		3 (75.0%)	1 (25.0%)	
Age	years old (means ± SD)	59.20 ± 11.34	62.14 ± 6.91	0.536	60.00 ± 13.04	60.23 ± 8.00	0.959	60.56 ± 11.15	59.00 ± 7.13	0.754
Sex	Male	9 (69.2%)	4 (30.8%)	1.000	5 (38.5%)	8 (61.5%)	1.000	9 (69.2%)	4 (30.8%)	1.000
	Female	6 (66.7%)	3 (33.3%)		4 (44.4%)	5 (55.6%)		7 (77.8%)	2 (22.2%)	
Site	Colon	3 (60.0%)	2 (40.0%)	1.000	3 (60.0%)	2 (40.0%)	1.000	5 (100%)	0 (0.0%)	0.266
	Rectum	12 (70.6%)	5 (29.4%)		6 (35.3%)	11 (64.7%)		11 (64.7%)	6 (35.3%)	
cT	T2+T3	5 (62.5%)	3 (37.5%)	1.000	2 (25.0%)	6 (75.0%)	0.380	6 (75.0%)	2 (25.0%)	1.000
	T4	10 (71.4%)	4 (28.6%)		7 (50.0%)	7 (50.0%)		10 (71.4%)	4 (28.6%)	
cN	N0	0 (0.0%)	0 (0.0%)	NA	0 (0.0%)	0 (0.0%)	NA	0 (0.0%)	0 (0.0%)	NA
	N1+N2	15 (68.2%)	7 (31.8%)		9 (40.9%)	13 (59.1%)		16 (72.7%)	6 (27.3%)	
cM	M0	0 (00.0%)	0 (00.0%)	NA	0 (00.0%)	0 (00.0%)	NA	0 (0.0%)	0 (0.0%)	NA
	M1	15 (68.2%)	7 (31.8%)		9 (40.9%)	13 (59.1%)		16 (72.7%)	6 (27.3%)	
cTNM	I+II	0 (0.0%)	0 (0.0%)	NA	0 (0.0%)	0 (0.0%)	NA	0 (0.0%)	0 (0.0%)	NA
	III+IV	15 (68.2%)	7 (31.8%)		9 (40.9%)	13 (59.1%)		16 (72.7%)	6 (27.3%)	
ypT	T1+T2+T3	8 (61.5%)	5 (38.5%)	0.648	6 (46.2%)	7 (53.8%)	0.674	6 (46.2%)	7 (53.8%)	0.333
	T4	7 (77.8%)	2 (22.2%)		7 (77.8%)	2 (22.2%)		3 (33.3%)	6 (66.7%)	
ypN	N0	5 (71.4%)	2 (28.6%)	1.000	3 (42.9%)	4 (57.1%)	1.000	4 (57.1%)	3 (42.9%)	0.334
	N1+N2	10 (66.7%)	5 (33.3%)		6 (40.0%)	9 (60.0%)		12 (80.0%)	3 (20.0%)	
ypM	M0	1 (100.0%)	0 (0.0%)	1.000	1 (100.0%)	0 (0.0%)	0.409	1 (100.0%)	0 (0.0%)	1.000
	M1	14 (70.0%)	6 (30.0%)		8 (38.1%)	13 (61.9%)		15 (71.4%)	6 (28.6%)	
ypTNM	I+II	1 (100.0%)	0 (0.0%)	1.000	1 (100.0%)	0 (0.0%)	0.409	1 (100.0%)	0 (0.0%)	1.000
	III+IV	14 (66.7%)	7 (33.3%)		8 (38.1%)	13 (61.9%)		15 (71.4%)	6 (28.6%)	
CSD	No	6 (85.7%)	1 (14.3%)	0.350	4 (57.1%)	3 (42.9%)	0.376	6 (85.7%)	1 (14.3%)	0.616
	Yes	9 (60.0%)	6 (40.0%)		5 (33.3%)	10 (66.7%)		10 (66.7%)	5 (33.3%)	
Death	No	6 (85.7%)	1 (14.3%)	0.350	4 (57.1%)	3 (42.9%)	0.376	6 (85.7%)	1 (14.3%)	0.616
	Yes	9 (60.0%)	6 (40.0%)		5 (33.3%)	10 (66.7%)		10 (66.7%)	5 (33.3%)	
Recurrence	No	1 (50.0%)	1 (50.0%)	1.000	9 (45.0%)	11 (55.0%)	0.494	14 (70.0%)	6 (30.0%)	1.000
	Yes	14 (70.0%)	6 (30.0%)		0 (0.0%)	2 (100.0%)		2 (100.0%)	0 (0.0%)	
Regression	Good	0 (0.0%)	0 (0.0%)	NA	0 (0.0%)	0 (0.0%)	NA	0 (0.0%)	0 (0.0%)	NA

	Poor	15 (68.2%)	7 (31.8%)		9 (40.9%)	13 (59.1%)		16 (72.7%)	6 (27.3%)	
Surgery	Curative	3 (60.0%)	2 (40.0%)	1.000	2 (40.0%)	3 (60.0%)	1.000	5 (100.0%)	0 (0.0%)	0.266
	Palliative	12 (70.6%)	5 (29.4%)		7 (41.2%)	10 (58.8%)		11 (64.7%)	6 (35.3%)	

ITB, intratumoral budding; DR, desmoplastic reaction; KM, Klintrup–Mäkinen grade; PBS, pretreatment biopsy sample; CRC, colorectal carcinoma; TSR, tumor stromal ratio; FOLFIRI, leucovorin, 5-fluorouracil and irinotecan combination; FOLFOX, leucovorin, 5-fluorouracil and oxaliplatin combination; c-, clinical; T, T category; N, N category; M, M category; TNM, tumor-node-metastasis stage; yp-; post-treatment pathologic ; CSD, cancer-specific death, NA, not available. Note. Because of all cases are classified into high TSR, the association of TSR and clinicopathological factors cannot calculated.

Table S5. The prognostic significances of the TME factors, such as ITB, DR, TSR, and KM grade in PBSs using the Kaplan-Meier curve analysis in two subgroups (CCRT vs. CTx).

	CCRT			CTx
	DFS (P value)	CSS (*P* value)	OS (*P* value)	CSS = OS (*P* value)
Age	0.611	0.783	0.164	0.687
Sex	0.29	0.214	0.418	0.491
cT	0.049	0.152	0.387	0.217
cN	0.874	0.218	0.150	NA
cM	0.941	0.482	0.771	NA
cTNM	0.874	0.218	0.150	NA
ITB	<0.001	0.006	0.068	0.107
DR	0.522	0.740	0.856	0.650
TSR	0.112	0.209	0.206	NA
KM	0.907	0.870	0.488	0.812

TME, tumor microenvironment; ITB, intratumoral budding; DR, desmoplastic reaction; TSR, tumor–stroma ratio; KM, Klintrup–Mäkinen grade; PBS, pretreatment biopsy sample; CRC, colorectal carcinoma; CCRT, concurrent chemoradiation therapy; CTx, chemotherapy, c-, clinical; T, T category; N, N category; M, M category; TNM, tumor-node-metastasis stage; DFS, disease-free survival; OS, overall survival; CSS, cancer-specific survival; NA, not available.

Table S6. Multivariate Cox proportional hazards analysis of predictors for CSS in the CCRT subgroup.

	CSS	
	Hazard ratio (95% CI)	*P* value
cTNM	0.533 (0.193-1.476)	0.226
ITB	3.545 (1.437-8.750)	0.006
DR	0.961 (0.357-2.588)	0.938
KM	0.849 (0.321-2.248)	0.742
TSR	0.468 (0.166-1.320)	0.151

cTNM, tumor-node-metastasis stage; CSS, cancer-specific survival; CCRT, concurrent chemoradiation therapy; TME, tumor microenvironment; ITB, intratumoral budding; DR, desmoplastic reaction; TSR, tumor–stroma ratio; KM, Klintrup–Mäkinen grade; cTNM, clinical tumor-node-metastasis stage.
